# Low initial body fat percentage exacerbates skeletal muscle loss and increases the risk of hyperuricemia during high-altitude acclimatization in young men

**DOI:** 10.3389/fnut.2026.1781965

**Published:** 2026-03-04

**Authors:** Yanlin Zhu, Jie He, Shuang Li, Jie Zhang, Huichang Jia, Yongjian Yang, Yi Li, Xianglian Li, Jian Li, Yunming Li, Yue Cheng

**Affiliations:** 1 Department of Nephrology, General Hospital of Western Theater Command, PLA, Chengdu, China; 2 Department of Cardiology, General Hospital of Western Theater Command, PLA, Chengdu, China; 3 Office of Medical Information and Data, Medical Support Center, General Hospital of Western Theater Command, PLA, Chengdu, China

**Keywords:** high altitude, hyperuricemia, initial body fat percentage, skeletal muscle mass, young men

## Abstract

**Background:**

The incidence of hyperuricemia (HUA) increases significantly after individuals ascend to a high-altitude environment, particularly among young men. Previous studies have shown correlations between skeletal muscle mass, fat mass, and serum uric acid levels. However, whether changes in body composition under high-altitude conditions influence the occurrence of HUA has not been reported.

**Objectives:**

This study aimed to investigate the effects of initial body fat percentage (BF%) and changes in body composition on the development of HUA in young men after 1 year at high altitude.

**Methods:**

In this prospective observational study, 216 young men who relocated from a plain area to a high-altitude area for 1 year were enrolled. Data on basic information, body composition, and laboratory measurements were collected both before relocation and after the one-year period.

**Results:**

After 1 year at high altitude, skeletal muscle mass (SMM) decreased significantly. Linear regression analysis revealed that the change in SMM was correlated with the initial BF%; a lower initial BF% was associated with a more pronounced decrease in SMM (*b* = 0.186, *p* < 0.001). A total of 136 participants (62.96%) were diagnosed with HUA after 1 year. Binary logistic regression analysis revealed high altitude (OR = 2.217, *p* < 0.05), low SMM (OR = 0.920, *p* < 0.05), low initial BF% (4–8%: OR = 3.142, *p* < 0.05; ≤4%: OR = 4.489, *p* < 0.01), and a decrease in SMM (−1.5 to −2.5 kg: OR = 2.599, *p* < 0.05; ≤−2.5 kg: OR = 3.263, *p* < 0.05) as risk factors for HUA. After adjusting for altitude and SMM, a decrease in SMM (−1.5 to −2.5 kg: OR = 2.735, *p* < 0.05; ≤−2.5 kg: OR = 3.198, *p* < 0.05) and low initial BF% (4–8%: OR = 2.687, *p* < 0.05; <4%: OR = 3.708, *p* < 0.01) remained predictive factors for HUA.

**Conclusion:**

Our findings indicate that for young men moving from plains to high altitudes, the BF% and the change in SMM can be used to predict the incidence of hyperuricemia after 1 year.

## 1 Introduction

The prevalence of HUA increases significantly in high-altitude environments, with young men being particularly susceptible (1, 2). The exact mechanism is not fully understood but may involve alterations in uric acid production and excretion due to hypoxic conditions. Serum uric acid is the end product of purine metabolism, and muscle is a primary source of purines. Multiple studies have reported correlations between muscle mass, its changes, and serum uric acid levels (3, 4). A previous study indicated that muscle mass is a major determinant of serum uric acid levels in male children and adolescents (5). Other studies report that xanthine oxidoreductase, which is responsible for uric acid production, is abundant in adipose tissue and that its enzyme activity increases with fat mass, suggesting that fat content may also influence serum uric acid levels (6, 7).

Upon ascending to high altitudes, factors such as hypoxia, increased basal metabolic rate, decreased appetite, and changes in physical activity lead the body into a state of negative energy balance. During a prolonged negative energy balance, stored energy (from glycogen, fat, and skeletal muscle protein) is sequentially consumed to meet metabolic demands (8). Because humans require a minimal essential fat content (4–8% for males, 8–19% for females), fat stores cannot be depleted entirely. Therefore, during sustained negative energy balance, the body relies on protein catabolism to meet energy needs (9, 10). Hypoxia-induced dysregulation of mTORC1 and upregulation of calpain- and ubiquitin–proteasome-mediated proteolysis may drive muscle protein catabolism in individuals moving from lowlands to high altitude, leading to loss of total body mass and fat-free mass (11). Body fat may attenuate muscle catabolism in physically active individuals in cold, high-altitude environments (8).

We hypothesize that, in addition to traditional risk factors (e.g., sex, age, ethnicity, BMI, diabetes, and hyperlipidemia) and high-altitude-related factors (1, 2, 12–15), changes in body composition may be associated with an increased risk of HUA in high-altitude environments. The aims of this study were to investigate the impact of the initial BF% and changes in body composition after 1 year in a high-altitude environment on HUA, identify risk factors for HUA among young men, and contribute to the development of better health plans for this population.

## 2 Methods

### 2.1 Study design and research subjects

This prospective observational study recruited 522 young men (aged 19–27) who were scheduled to move from a plain area (500 m) to an ultrahigh altitudes area (3,500–5,500 m) in May 2022. Data on basic information, body composition, and laboratory tests were collected before relocation. In May 2023, data were collected again after 1 year at high altitude. A total of 312 participants completed the follow-up examinations. After 56 participants who were lost to follow-up, 28 with incomplete clinical data, and 12 with preexisting conditions (renal insufficiency, HUA, hyperlipidemia, diabetes, or other metabolic diseases) were excluded, 216 participants were ultimately included. All participants were of Han ethnicity. Participants followed a standardized, low-volume exercise protocol to minimize interindividual variance in energy expenditure, including performing 30 min of steady-state walking on a motorized treadmill, 2–3 days per week. Exercise intensity was prescribed on an individual basis using heart rate reserve. Participants were instructed to refrain from any additional structured exercise. All participants consumed standardized meals from the institutional canteen throughout the study. To minimize variability in purine intake before venous blood sampling, a 3-day low-purine dietary intervention was strictly enforced. This included specialized menus free of high-purine ingredients, developed by clinical nutritionists. Written informed consent was obtained from all participants, and the study was approved by the Ethics Committee of the Western Theater General Hospital (2022EC3-ky062).

### 2.2 Data collection and anthropometry

Demographic and health-related information was collected using standardized questionnaires. All the subjects received training to ensure accurate data collection and measurement capabilities. The information collected included demographic characteristics (sex, age) and personal medical history (kidney disease, metabolic diseases, cardiovascular diseases). Standing height was measured using a fixed stadiometer. The altitude was recorded using GPS. Body weight, fat mass, skeletal muscle mass, body fat percentage, and water content were measured in the morning under overnight-fasted conditions using bioelectrical impedance analysis (InBody S10, Biospace Inc., South Korea). The average of three blood pressure (BP) readings was recorded using a digital BP monitor. Body mass index (BMI) was calculated as weight (kg) divided by height (m) squared (kg/m^2^).

### 2.3 Laboratory measurements

After fasting for at least 8 h, venous blood samples were collected. Data, including serum creatinine, serum uric acid, blood lipid, and fasting blood glucose levels, were obtained. All venous blood samples were analyzed by the KingMed Diagnostics Laboratory Center.

### 2.4 Definitions

Hyperuricemia (HUA) was defined as a serum uric acid concentration ≥420.0 μmol/L in males (16).

### 2.5 Statistical methods

Statistical analyses were performed using IBM SPSS Statistics 26.0. Normally distributed continuous variables are presented as the mean ± standard deviation (SD), nonnormally distributed continuous variables are presented as the median (P25, P75), and categorical variables are presented as the frequency and percentage. Comparisons of normally distributed variables between the same participants at the plain and after 1 year at high altitude were made using paired-sample t tests; nonnormally distributed variables were compared using the Wilcoxon signed-rank test. Comparisons between the HUA and non-HUA groups after 1 year for normally distributed variables were performed using independent-sample t tests; for nonnormally distributed variables, the Mann–Whitney U test was used; and for categorical variables, Pearson’s chi-square test or Fisher’s exact test was used. The relationship between the initial BF% and the change in SMM was analyzed using linear regression. The influence of different body composition parameters and their changes on HUA was analyzed using binary logistic regression models: Model 1 was unadjusted, Model 2 was adjusted for altitude, and Model 3 was further adjusted for SMM based on Model 2. Risk factors for HUA related to body composition after moving from the plain to high altitude were identified, and odds ratios (ORs) and 95% confidence intervals (CIs) were calculated. A *p* value < 0.05 was considered to indicate statistical significance.

## 3 Results

### 3.1 Comparison of related indicators and body composition at the plain and after 1 year at high altitude

This study included 216 young men (aged 19–27). Compared with plain levels, significant changes in body water, SMM, fat mass, BMI, creatinine, and uric acid were observed after 1 year at high altitude (Table 1).

**Table 1:** Comparison of various indicators at the plain and after 1 year at high altitude.

Variables	All (*n* = 432)	Plain (*n* = 216)	High altitude (*n* = 216)	*t*/*z*	*p* value
BMI (kg/m^2^)	21.90 ± 2.24	22.02 ± 2.14	21.78 ± 2.32	1.212^b^	0.227
TBW (kg)	43.23 ± 4.51	44.41 ± 4.54	42.06 ± 4.17	18.315^b^	<0.001
BF%	7.80 (5.00, 10.50)	7.30 (3.80, 9.95)	8.40 (5.80, 11.10)	5.953^c^	<0.001
Fat (kg)	5.15 (3.20, 6.90)	4.80 (2.50, 6.80)	5.45 (3.80, 7.38)	5.313^c^	<0.001
SMM (kg)	33.46 ± 3.72	34.05 ± 3.68	32.87 ± 3.67	9.952^b^	<0.001
UA (μmol/L)	414.15 ± 83.84	374.39 ± 66.82	453.90 ± 78.62	12.547^b^	<0.001
Cr (μmol/L)	81.88 ± 10.33	76.36 ± 8.05	87.41 ± 9.36	13.092^b^	<0.001

^a^*χ*^2^ test; ^b^Paired samples t-test; ^c^Wilcoxon-Mann–Whitney test; BMI, body mass index; TBW, total body water; UA, uric acid; Cr, creatinine; BF%, body fat percentage; SMM, skeletal muscle mass.

### 3.2 Comparison of indicators between the HUA and non-HUA groups after 1 year at high altitude

After 1 year at high altitude, 136 out of 216 subjects (62.96, 95% CI [56.52, 69.40%]) were diagnosed with HUA. Compared with the non-HUA group, the HUA group had higher altitude levels, lower SMM, a lower initial BF%, and a more significant decrease in SMM, with all the differences being statistically significant (Table 2).

**Table 2:** Comparison of various indicators between the non-hyperuricemia group and the hyperuricemia group.

Variables	All (*n* = 216)	Non-HUA (*n* = 80)	HUA (*n* = 136)	*χ*^2^/*t*/*z*	*p* value
Age (years)	21.99 ± 1.45	21.90 ± 1.35	22.04 ± 1.51	0.073^b^	0.483
Altitude (km)	3.99 ± 0.41	3.91 ± 0.36	4.03 ± 0.44	2.168^b^	0.031
BMI (kg/m^2^)	21.78 ± 2.32	22.00 ± 2.52	21.65 ± 2.20	1.073^b^	0.284
TBW (kg)	42.06 ± 4.17	41.85 ± 4.18	42.18 ± 4.18	0.564^b^	0.573
Fat (kg)	5.45 (3.80, 7.38)	5.40 (3.85, 6.98)	5.55 (3.80, 7.48)	0.294^c^	0.769
SMM (kg)	32.87 ± 3.67	33.57 ± 3.83	32.46 ± 3.52	2.157^b^	0.032
Cr (μmol/L)	87.41 ± 9.36	85.86 ± 8.78	88.33 ± 9.60	1.886^b^	0.061
Initial BF%					
≥12	32 (14.81)	19 (59.38)	13 (40.62)	12.136^a^	0.007
8–12	64 (29.63)	27 (42.19)	37 (57.81)		
4–8	63 (29.17)	20 (31.75)	43 (68.25)		
≤4	57 (26.39)	14 (24.56)	43 (75.44)		
Fat change (kg)					
≥2.5	47 (21.76)	13 (27.66)	34 (72.34)	4.474^a^	0.483
1.5–2.5	45 (20.83)	15 (33.33)	30 (66.67)		
0.5–1.5	41 (18.98)	15 (36.59)	26 (63.41)		
−0.5 to 0.5	32 (14.81)	13 (40.62)	19 (59.38)		
−0.5 to −1.5	20 (9.26)	9 (45.00)	11 (55.00)		
≤−1.5	31 (14.35)	15 (48.39)	16 (51.61)		
SMM change (kg)					
≥1.5	12 (5.56)	9 (75.00)	3 (25.00)	28.853^a^	<0.001
0.5–1.5	27 (12.50)	18 (66.67)	9 (33.33)		
−0.5 to 0.5	39 (18.06)	17 (43.59)	22 (56.41)		
−0.5 to −1.5	43 (19.91)	16 (37.21)	27 (62.79)		
−1.5 to −2.5	48 (22.22)	11 (22.92)	37 (77.08)		
≤−2.5	47 (21.76)	9 (19.15)	38 (80.85)		

^a^*χ*^2^ test; ^b^Independent samples t-test; ^c^Mann–Whitney U test; HUA, hyperuricemia; BMI, body mass index; TBW, total body water; UA, uric acid; Cr, creatinine; BF%, body fat percentage; SMM, skeletal muscle mass.

### 3.3 Impact of initial body fat percentage on changes in skeletal muscle mass after entering high altitude

**Figure 1:**
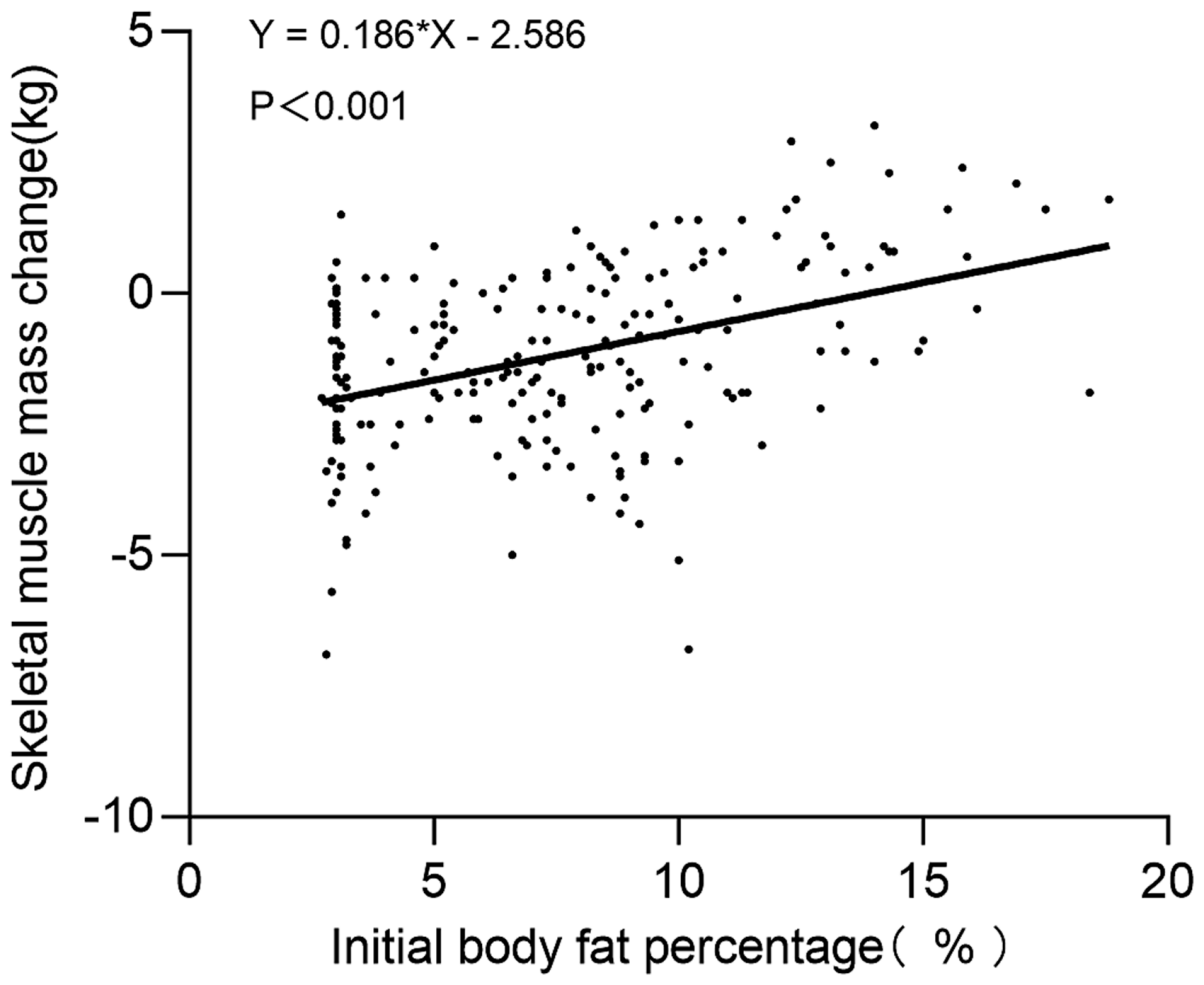
Correlation between initial body fat percentage and skeletal muscle mass change following high-altitude acclimatization. Each point represents an individual participant (*n* = 216). The line shows linear regression fit.

Linear regression analysis revealed a significant correlation between initial BF% and the change in SMM after 1 year at high altitude (*b* = 0.186, 95% CI [0.130, 0.242], *p* < 0.001). Subjects with a lower initial BF% experienced a more pronounced decrease in SMM after entering high altitude (Figure 1).

### 3.4 Body composition parameters and the risk of hyperuricemia

The binary logistic regression results are shown in Table 3; high altitude (OR = 2.217, *p* < 0.05), low SMM (OR = 0.920, *p* < 0.05), low initial BF% (4–8%: OR = 3.142, *p* < 0.05; ≤4%: OR = 4.489, *p* < 0.01), and a decrease in SMM (−1.5 to −2.5 kg: OR = 2.599, *p* < 0.05; ≤−2.5 kg: OR = 3.263, *p* < 0.05) were risk factors for HUA. After adjusting for altitude and SMM, a decrease in SMM (−1.5 to −2.5 kg: OR = 2.735, *p* < 0.05; ≤−2.5 kg: OR = 3.198, *p* < 0.05) and low initial BF% (4–8%: OR = 2.687, *p* < 0.05; <4%: OR = 3.708, *p* < 0.01) remained predictive factors for HUA (Table 3).

**Table 3:** Logistic regression analysis of the risk of hyperuricemia.

Variables	OR (95% CI)		
	Model 1	Model 2	Model 3
Altitude (km)	2.217 (1.063, 4.623)^*^	/	/
SMM (kg)	0.920 (0.852, 0.994)^*^	0.931 (0.861, 1.006)	/
Initial BF%			
≥12	1	1	1
8–12	2.003 (0.845, 4.745)	1.848 (0.774, 4.413)	1.790 (0.747, 4.293)
4–8	3.142 (1.300, 7.596)^*^	2.865 (1.175, 6.982)^*^	2.687 (1.092, 6.609)^*^
≤4	4.489 (1.774, 11.356)^**^	4.157 (1.633, 10.580)^**^	3.708 (1.422, 9.665)^**^
SMM change (kg)			
≥1.5	0.258 (0.060, 1.100)	0.297 (0.068, 1.289)	0.315 (0.072, 1.380)
0.5–1.5	0.386 (0.139, 1.072)	0.440 (0.156, 1.246)	0.463 (0.162, 1.322)
−0.5 to 0.5	1	1	1
−0.5 to −1.5	1.304 (0.538, 3.159)	1.465 (0.592, 3.625)	1.449 (0.585, 3.591)
−1.5 to −2.5	2.599 (1.032, 6.548)^*^	2.718 (1.071, 6.893)^*^	2.735 (1.076, 6.952)^*^
≤−2.5	3.263 (1.245, 8.552)^*^	3.319 (1.257, 8.766)^*^	3.198 (1.205, 8.487)^*^

Model 1: Crude model.

Model 2: Adjusted for altitude.

Model 3: Adjusted for altitude and SMM.

*p < 0.05, **p < 0.01. OR, odds ratio; CI, confidence interval; BF%, body fat percentage; SMM, skeletal muscle mass.

## 4 Discussion

This study revealed changes in serum uric acid levels and body composition in 216 healthy young men 1 year after they relocated from a plain to a high-altitude area. Serum uric acid levels increased significantly in this cohort after 1 year, with 136 cases (62.96%) of HUA. SMM decreased significantly after 1 year. Further analysis revealed a correlation between the initial BF% and the extent of SMM loss; a lower initial BF% was associated with a greater decrease in SMM. Binary logistic regression analysis indicated that a decrease in SMM and a low initial BF% were risk factors for HUA after 1 year. The risk of HUA increased progressively when SMM loss exceeded 1.5 kg and when the initial BF% was less than 8%.

Numerous previous studies have established that the prevalence of HUA is greater among people of Han ethnicity on the Tibetan Plateau than among indigenous Tibetans, with higher rates in males than in females, and the prevalence in adult males decreases with age (1, 2). Therefore, this study focused on young males, a group with a high prevalence rate. A one-year period was chosen to ensure participants had progressed beyond acute acclimatization, allowing body composition and metabolic parameters to reflect chronic adaptive states (17).

Ascending to high altitude leads to changes in body composition due to hypoxia, increased energy expenditure, and changes in physical activity. Acclimatization to hypoxia and altitude related illness increases energy expenditure, which can lead to a 17–27% increase in the basal metabolic rate (18, 19). Energy expenditure exceeds energy intake, resulting in a negative energy balance, which leads to a reduction in body mass from fat and muscle mass. Previous research has reported that in cold, high-altitude environments, muscle mass decreases in men, whereas fat mass does not significantly change, and compared with individuals with a higher initial BF%, individuals with a lower initial BF% lose more muscle mass (8). Other studies suggest that hypoxia-mediated upregulation of proteolysis may drive muscle protein catabolism in individuals moving from lowlands to high altitude, leading to loss of total body mass and fat-free mass (11). Our study revealed a significant decrease in SMM after 1 year, which correlated with the initial BF%; the lower the initial BF% was, the greater the loss. This is likely because high-altitude environments often induce a negative energy balance. Young males generally have a lower BF%, and the body has limited usable fat stores, necessitating increased reliance on muscle protein catabolism for energy. Due to limitations in the study conditions, this research did not observe objective indicators such as physical activity level, dietary macronutrient content, partial pressure of oxygen, oxygen saturation, pulmonary function, or cardiac function, and thus lacks objective evidence of hypoxia and negative energy balance. Therefore, our hypothesis regarding negative energy balance is merely speculative.

Approximately 80% of uric acid in the body originates from endogenous purine metabolism, and muscle protein is considered the largest source of purines in the body (20). Exhaustion of muscle cells and ATP metabolism release large amounts of nucleic acids and purines, leading to increased production of serum uric acid (21). The relationship between serum uric acid levels and SMM has been debated across different ages and sexes. Some studies have shown that HUA is associated with low SMM in middle-aged and elderly people (22), whereas others have suggested a link between higher uric acid levels and better muscle function in this group (23, 24). Another study revealed sex differences in uric acid levels beginning at puberty, when renal uric acid excretion decreases and muscle mass increases significantly, especially in boys, leading to rising uric acid levels (5). Our study revealed that a decrease in SMM exceeding 1.5 kg after entering high altitude was a risk factor for HUA. The risk was 2.735 times greater for those who lost 1.5–2.5 kg of SMM and 3.198 times greater for those who lost >2.5 kg than for those with no significant change, suggesting that increased skeletal muscle catabolism may contribute to elevated uric acid levels in young men at high altitude. Although lower absolute SMM was associated with a higher HUA risk in unadjusted analyses, it lost significance after adjusting for altitude, SMM change, and initial BF%, indicating that the change in SMM after ascent has a greater effect on uric acid levels than the absolute value does.

Initial BF% was the strongest predictor of HUA risk. The risk for subjects with an initial BF% of 4–8% was 2.687 times greater, and for those with ≤4%, it was 3.708 times greater than that for those with ≥12%. We speculate that a lower initial BF% might influence uric acid levels through two pathways: (1) by increasing skeletal muscle protein catabolism, thereby increasing uric acid production, and (2) by leading to greater consumption of carbohydrates and muscle protein for energy, potentially preserving or even increasing fat mass, which could indirectly affect uric acid levels. Previous studies have shown that increased fat mass can reduce renal uric acid clearance and increase xanthine oxidase activity in adipose tissue, increasing serum uric acid levels (25, 26). In our cohort, overall fat mass increased at high altitude compared with that at low altitude, and the increase was greater in the HUA group than in the non-HUA group, although the difference was not statistically significant. This potential influence of fat mass cannot be ignored. Previous research has also suggested that the combination of decreased SMM and increased fat mass may create a dual metabolic burden, increasing the risk of metabolic diseases (27).

Substantial evidence links altitude, BMI, and age to uric acid levels or HUA risk (28). Our unadjusted model also identified altitude as a risk factor, but its effect became nonsignificant after adjusting for muscle mass and other factors. Owing to the narrow age range (19–27 years) of young men and the normal BMI values in our cohort, no significant effects of age or BMI on HUA were found.

This study has several limitations. First, the sample size was relatively small, posing a risk of sampling error. Second, this study restricted the participants to Han males aged 19–27 years and was conducted exclusively in ultrahigh altitude areas (3,500–5,500 m), which limits the generalizability of the findings to broader populations (including females, other age groups, and different ethnic groups) and to other altitude categories. Third, no intermediate assessments were conducted throughout the year to document the chronology of physiological and body composition changes, and larger prospective clinical studies incorporating serial assessments to characterize the time course are needed. Fourth, while participants adhered to a standardized low-purine diet, we did not collect quantitative data on total caloric intake, macronutrient distribution, dietary patterns, or water consumption. Individual variations in energy balance and hydration status could represent unmeasured confounders that influence serum urate levels and body composition. Future high-altitude studies would benefit from detailed dietary records and hydration monitoring to disentangle the specific contributions of nutritional factors. Fifth, the absence of hematocrit measurements means that the confounding effect of hemoconcentration on uric acid levels cannot be ruled out. This feedback underscores the need for future studies to develop and validate diagnostic standards appropriate for high-altitude settings.

## 5 Conclusion

In conclusion, this observational study of young men ascending to high altitude revealed a high prevalence of HUA in this population. The initial body fat percentage significantly influenced the incidence of HUA after 1 year, and changes in skeletal muscle mass likely played an important mediating role. Therefore, in clinical practice, attention should be given to the initial BF% and changes in SMM for individuals moving from the plains to high altitudes. Appropriately increasing the BF% before ascent might help reduce the risk of developing HUA among people who stay on the plateau for a year, suggesting a potential preventive target for high-altitude hyperuricemia. However, this observation must be balanced against the established association between higher adiposity and increased risk of acute mountain sickness (AMS). Future studies are warranted to delineate the optimal BF% range that confers urate-lowering benefit without compromising altitude tolerance. Furthermore, larger prospective studies are necessary to determine whether increasing the initial BF% can reduce the loss of SMM and lower serum uric acid levels. The underlying mechanisms linking body composition and uric acid metabolism require further in-depth investigation.

## Data Availability

The raw data supporting the conclusion of this article is not publicly available due to privacy/ethical restrictions. Requests to access the datasets should be directed to the corresponding author (YC).
